# Linkage to care, mobility and retention of HIV‐positive postpartum women in antiretroviral therapy services in South Africa

**DOI:** 10.1002/jia2.25114

**Published:** 2018-07-19

**Authors:** Tamsin K Phillips, Kate Clouse, Allison Zerbe, Catherine Orrell, Elaine J Abrams, Landon Myer

**Affiliations:** ^1^ Division of Epidemiology & Biostatistics Centre for Infectious Disease Epidemiology & Research School of Public Health & Family Medicine University of Cape Town Cape Town South Africa; ^2^ The South African Department of Science and Technology/National Research Foundation (DST‐NRF) Centre of Excellence in Epidemiological Modelling and Analysis (SACEMA) Stellenbosch University Stellenbosch South Africa; ^3^ Vanderbilt Institute for Global Health Vanderbilt University Nashville TN USA; ^4^ Department of Medicine Division of Infectious Diseases Vanderbilt University Nashville TN USA; ^5^ ICAP Columbia University Mailman School of Public Health New York NY USA; ^6^ Desmond Tutu HIV Centre Institute of Infectious Disease and Molecular Medicine and Department of Medicine University of Cape Town Cape Town South Africa; ^7^ College of Physicians & Surgeons Columbia University New York NY USA

**Keywords:** antiretroviral therapy, postpartum, mobility, transfer, linkage, retention, viral suppression, South Africa

## Abstract

**Introduction:**

Linkage to care and mobility postpartum present challenges to long‐term retention after initiating antiretroviral therapy (ART) in pregnancy, but there are few insights from sub‐Saharan Africa. We aimed to describe postpartum linkage to care, mobility, retention and viral suppression after ART initiation in pregnancy.

**Methods:**

Using routine electronic data we assessed HIV‐specific health contacts and clinic movements among women initiating ART in an integrated antenatal care (ANC) and ART clinic in Cape Town, South Africa. The local care model includes mandatory transfer to general ART clinics postpartum. We investigated *linkage to care* after leaving the integrated clinic and *mobility* to new clinics until 30 months on ART. We used Poisson regression to explore predictors of linkage, *retention* (accessing care at least once at both 12 [6 to <18] and 24 [18 to <30] months on ART), and *viral suppression* (HIV viral load [VL] ≤50 and ≤1000 copies/mL after 12 months on ART).

**Results:**

Among 617 women, 23% never linked to care; 71% and 65% were retained at 12 and 24 months on ART respectively, with 59% retained in care at both times. Those who linked (n = 485) accessed HIV care at 98 different clinics and 21% attended ≥2 clinics. Women >25 years, married/cohabiting or presenting early for ANC were more likely to link. Younger and unemployed women were more likely to attend ≥2 clinics (adjusted risk ratio [aRR] 1.10 95% confidence interval [CI] 1.02 to 1.18 and aRR 1.06 95% CI 0.99 to 1.12 respectively). Age >25 years (aRR 1.17 95% CI 1.02 to 1.33) and planned pregnancy (aRR 1.20 95% CI 1.09 to 1.33) were associated with being retained. Among 338 retained women with VL available, attending ≥2 clinics reduced the likelihood of viral suppression when defined as ≤50 copies/mL (aRR 0.81 95% CI 0.69 to 0.95). Distance moved was not associated with VL.

**Conclusions:**

These data show that a substantial proportion of women do not link to postpartum ART care in this setting and, among those that do, long‐term retention remains a challenge. Women move to a variety of clinics and young women appear particularly vulnerable to attrition. Interventions promoting linkage and continued retention for women initiating ART during pregnancy warrant urgent consideration.

## 1 Introduction

Population movement has received much attention in the context of the HIV epidemic 1, 2. Migration and mobility may be associated with HIV acquisition and providing HIV care to mobile populations presents particular challenges 3, 4, 5, 6. In South Africa, movement between rural and urban areas for employment, education, healthcare, cultural and family reasons occurs frequently, involving all demographic groups, including women of reproductive age 7, 8, 9.

Antiretroviral therapy (ART) during pregnancy and breastfeeding, and associated viral suppression, reduces mother‐to‐child transmission (MTCT), improves maternal health, and reduces sexual transmission 10. However, these benefits hinge on women initiating ART, adhering to treatment, and remaining in care in the long term. Postpartum retention is a major challenge and there is an urgent need to understand how mobility may contribute to this 11, 12, 13, 14, 15.

In urban South Africa as well as other settings in sub‐Saharan Africa, pregnant HIV‐positive women who are not yet on ART start treatment during pregnancy in integrated clinics providing antenatal care (ANC) and HIV care including ART. Time in the integrated clinic after delivery varies, but ultimately women must transfer their HIV care and link to general ART clinics postpartum. Additional movement between healthcare facilities also occur due to relocation and patient choice. These movements may introduce challenges to the continuum of HIV care and maternal health services 3, 4, 16. In South Africa, a recent analysis found that 38% of postpartum women who were considered lost to follow‐up (LTFU) at the clinic of ART initiation were in care elsewhere, and 33% received care outside of the province where they started ART 13. However, there are few data on the mobility of women with mandatory movement of ART care postpartum, and there is a need to understand the specific challenges related to linkage to care and mobility after delivery in these settings.

To address this, we explored continuity of care including linkage, geographic mobility and retention in care in a cohort of women who initiated ART in an integrated ANC‐ART clinic. The objectives were (i) to describe linkage to care after leaving the integrated clinic and additional mobility after linking and (ii) to explore whether frequency or distance of clinic movement were associated with outcomes of retention in care and, in a subset of women, viral suppression.

## 2 Methods

### 2.1 Setting

This is a secondary analysis of women enrolled into the Maternal & Child Health – Antiretroviral Therapy (MCH‐ART) study, which investigated optimal ART services for pregnant and postpartum women (ClinicalTrials.gov NCT01933477). This study was conducted at a large primary healthcare clinic in Cape Town, South Africa in an area with high rates of unemployment and poverty 17. ANC coverage is high (approximately 95%) and the antenatal HIV seroprevalence is approximately 30% 18. The clinic serves over 4000 women annually from a wide catchment area. Women from neighbouring areas of Cape Town as well as from other provinces are known to access services here 19.

ART initiation and follow‐up are provided with ANC by nurse‐midwives throughout pregnancy. During the study period, ART eligibility was based on local public‐sector guidelines (WHO stage III/IV disease or CD4 count ≤350 cells/μL until June 2013, and thereafter universal ART for pregnant women regardless of disease stage). All women initiated a fixed‐dose combination of efavirenz, emtricitabine and tenofovir, and initiation usually occurred within a week of presentation for ANC. Per local standard of care, all women were transferred out to general ART services after delivery. They were provided with up to 3 months’ supply of ART and a transfer letter to their new clinic, chosen based on preference or proximity to where a woman lived. Women were instructed to attend the new clinic before the end of her ART supply but no additional support for linkage occurs in this setting.

### 2.2 Data sources

Data for this analysis came from multiple sources. Retrospective data from available routine electronic data sources were assembled for all enrolled women through a minimum of 30 months on ART. Additional baseline data for all women, and for a subset of women additional prospectively collected data, were obtained from the parent study. The data sources are described in detail below.

The parent study methods have been described previously 20. Briefly, between April 2013 and June 2014, 628 ART‐eligible pregnant women were consecutively enrolled when they presented for ANC at the integrated clinic. Study measurement visits occurred prospectively through one month postpartum in all women and through 18 months postpartum in a subset of breastfeeding women (n = 471). Mandatory transfer out of the integrated clinic occurred at six weeks postpartum for most women per local standard of care. By study design, 233 women remained in the integrated clinic for up to 12 months postpartum (median 7 months, IQR 2 to 12). Data from the parent study provided details on baseline demographics, timing of ART initiation, delivery outcomes and last visit in the integrated clinic.

As part of the parent study, routine electronic health data were abstracted retrospectively through at least 30 months after ART initiation for all women (final data point December 2016). Data were abstracted from the National Health Laboratory Services (NHLS) database, which provides laboratory data for public health facilities in all provinces of South Africa. In addition, electronic data on pharmacy dispensing and clinic contacts, including facility recorded deaths were obtained from the Provincial Health Data Centre (PHDC) of the Western Cape Department of Health. These data are linked with a unique patient identifier and include all public health facilities in the Western Cape Province. Contacts at hospitals and other non‐HIV services were excluded.

### 2.3 Measures

We brought together the above data sources to measure the following constructs. First, we defined *linkage to care* after leaving the integrated clinic based on evidence of at least one HIV‐specific contact (routine ART clinic visit, antiretroviral (ARV) pharmacy refill or a CD4 cell count or HIV VL laboratory test) between the last visit at the integrated clinic and 30 months after ART initiation. Second, we assessed *mobility*, by determining the location and counting each clinic attended after leaving the integrated clinic. This was analysed as a binary variable of one *versus* ≥2 different clinics. Third, we created a global measure of *retention in HIV care* based on evidence of at least one HIV‐specific contact at both 12 (6 to <18) and 24 (18 to <30) months after ART initiation at any clinic (including the integrated clinic for any women who linked to care prior to 30 months on ART but had not been transferred out of the integrated clinic by 12 months on ART). In sensitivity analyses we also examined evidence of HIV‐specific contact at only 24 (18 to <30) months after ART initiation and at 18 (12 to <24) months postpartum. A 12‐month window was used in all definitions as, although routine ART visits and ARV dispensing are expected more regularly, routine HIV laboratory results (our only nationally available data source) are only expected annually in this setting. Fourth, among women considered to be retained in HIV care, we investigated HIV *viral suppression* based on any HIV RNA VL taken nearest to 24 months on ART and at least 12 months after ART initiation. These were primarily routine care VL results from the NHLS database. However, if no routine VL was found, available VL results from the MCH‐ART study were used. VLs were found for 338 women; 61% from NHLS. VL thresholds of ≤50 and ≤1000 copies/mL were used to define suppression based on definitions of suppression and flags for treatment failure in the South African National ART guidelines 21.

### 2.4 Analysis

Analyses were conducted in STATA 14 (STATA Corporation, College Station, TX). Descriptive analysis used frequencies and proportions, means with standard deviations (SD) or medians with interquartile ranges (IQR) with chi squared tests, Fisher's exact test, *t*‐tests or rank sum tests as appropriate. ArcMap 10.3.1 (Esri, Inc., Redlands, CA, USA) was used to describe the spatial distribution of continued care after the integrated clinic. Multivariable Poisson regression models with robust standard errors were used to estimate the relative risk of each outcome 22. Covariates that reached *p* < 0.10 in bivariate analyses were included in model building using a step‐wise approach. Although the parent MCH‐ART trial intervention was not the focus of this analysis, the MCH‐ART intervention did impact retention in HIV care at 12 months postpartum in the primary trial analysis 23 and some differences were seen for the retention outcomes in this analysis (Table S6). To account for differences in subgroups of women who received continued prospective follow‐up and/or delayed transfer out of the integrated clinic as part of the MCH‐ART study, all multivariable models were adjusted for design status in the MCH‐ART study in. Results are presented as crude or adjusted relative risks (RR or aRR) with 95% confidence intervals (CI). In this exploratory secondary analysis which may not have had sufficient power to detect small associations, all associations reaching *p* < 0.10 were discussed.

### 2.5 Ethics

All women included in this analysis completed written informed consent that included consent to review their routine medical records. Ethical approval was obtained from both the University of Cape Town Human Research Ethics Committee and the Columbia University Medical Centre Institutional Review Board.

## 3 Results

Among 628 women who initiated ART in pregnancy, eight women were found to have died and three to have relocated out of South Africa during the study period (up to 30 months on ART). These women were excluded from further analysis. Of the remaining 617 women, the mean age was 29 years (SD 5.3), 41% were married/cohabiting, 38% were employed and 26% had completed high school (Table 1). More than half the women (54%) were newly diagnosed with HIV in the incident pregnancy and 45% presented for ANC at ≤20 weeks gestation.

**Table 1:** Description of 617 HIV‐positive women who initiated ART during pregnancy, by linkage to HIV care after leaving the integrated clinic. Presented as n (%) unless specified

	Linked to care	Did not link to care	All women	*p*‐value
Number of women	485 (79)	132 (21)	617 (100)	
Median (IQR) months from ART initiation until last evidence of accessing care	28 (22 to 29)	4 (2 to 8)	26 (12 to 29)	<0.001
Characteristics at enrolment				
Mean age (SD)	29 (5.4)	28 (5.3)	29 (5.3)	0.015
Age ≤25	122 (25)	48 (37)	170 (28)	0.011
Married/cohabiting	216 (45)	37 (28)	253 (41)	0.001
Completed secondary school	124 (26)	39 (30)	163 (26)	0.358
Employed	182 (38)	52 (39)	234 (38)	0.695
First pregnancy	83 (17)	29 (22)	112 (18)	0.199
Intended pregnancy	146 (30)	35 (27)	181 (29)	0.422
Diagnosed with HIV in this pregnancy	255 (53)	81 (61)	336 (54)	0.072
Mean weeks gestation (SD)	21 (7.4)	23 (7.8)	21 (7.5)	0.005
Presented for ANC before 20 weeks gestation	231 (48)	47 (36)	278 (45)	0.014
Median (IQR) CD4 cell count at presentation for ANC (n = 601)	342 (235 to 509)	386 (253 to 555)	345 (236 to 513)	0.110
Characteristics at delivery				
Place of delivery (n = 597)				0.624
Delivered in primary care	190 (41)	56 (43)	246 (41)	
Delivered at tertiary hospital	277 (59)	74 (57)	351 (59)	
Delivery outcome				0.253
Live birth	464 (96)	131 (99)	595 (96)	
Stillbirth	11 (2)	1 (1)	12 (2)	
Miscarriage	6 (1)	0 (0)	6 (1)	
Unknown	4 (1)	0 (0)	4 (1)	

### 3.1 Linkage to care

**Figure 1:**
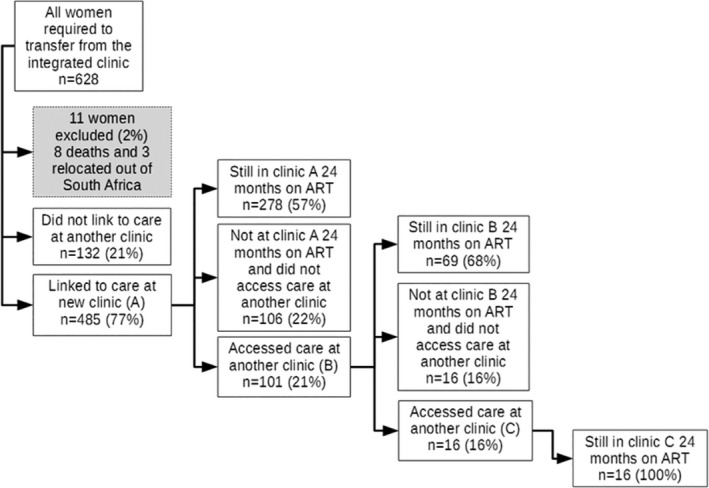
Flow of mobility for routine HIV care after leaving the integrated clinic through to 30 months after ART initiation.

Figure 1 describes the flow of access to HIV care after leaving the integrated clinic and Table 1 describes the characteristics among women who did and did not link to a new clinic after their last visit in the integrated clinic. There were 132 women (21%) with no evidence of linking to HIV care during the follow‐up period. Of these, nine women were not seen after their first ANC visit. Among the 485 women who did link to care, 384 (79%) had evidence of attending one clinic, 85 (18%) linked to two and 16 (3%) linked to three different clinics (Figure 1). There were 20 women who moved and linked to a new ART clinic while still pregnant, while the remaining 465 women linked to a new clinic after delivery.

Women who did not link to care during the follow‐up period were slightly younger (mean age 28 *vs*. 29), less likely to be married/cohabiting (28% *vs*. 45%) and more often diagnosed with HIV in this pregnancy (61% *vs*. 53%), compared to women who did link to care (Table 1). They also presented for ANC later (mean gestation 23 *vs*. 21 weeks). The associations between successful linkage and age >25 years (aRR 1.11 95% CI 1.00 to 1.23), being married/cohabiting (aRR 1.13 95% CI 1.04 to 1.23) and presentation for ANC ≤20 weeks gestation (aRR 1.11 95% CI 1.02 to 1.20) persisted in multivariable models (Table 2).

**Table 2:** Poisson regression model (n = 617) predicting whether a woman linked to HIV care at any other clinic after the integrated clinic. Presented as unadjusted (RR) and adjusted (aRR) risk ratios with 95% confidence intervals (CI)

	Crude		Adjusted	
	RR	95% CI	aRR	95% CI
Age >25	1.13	1.02 to 1.26	1.11	1.00 to 1.23
Married/cohabiting	1.16	1.07 to 1.25	1.13	1.04 to 1.23
Diagnosed with HIV in this pregnancy	0.93	0.85 to 1.01	–	
Presented for ANC ≤20 weeks	1.11	1.02 to 1.20	1.11	1.02 to 1.20

### 3.2 Mobility

After leaving the integrated clinic, women accessed care at 98 different clinics across South Africa, excluding the integrated clinic. The median distance moved for initial linkage was 1 kilometre (km) (IQR <1 to 3, maximum 1271 km) and 95% of clinics initially linked to were in the Western Cape Province. After linkage, an additional 117 movements were observed and the distance between clinics increased with additional moves. The median distance of second (n = 101) and third (n = 16) move was 3 km (IQR 1 to 108) and 413 km (IQR 1 to 945) respectively, with 23% of the second and 25% of the third move being out of the Western Cape Province.

**Figure 2:**
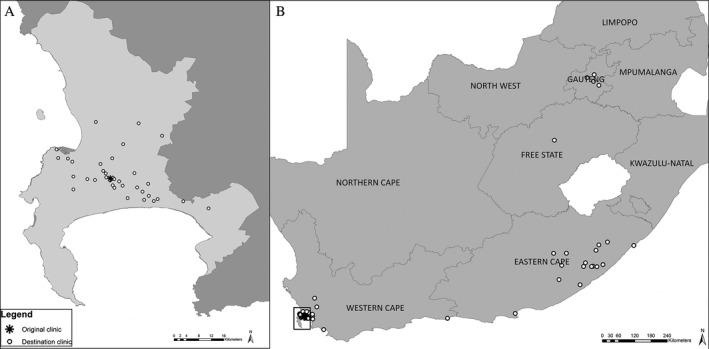
Maps of clinics attended for HIV care after leaving the integrated clinic **(A)** within the Cape Town Metropole and **(B)** within South Africa.

Overall, 270 women (56%) remained within the integrated clinic health district (clinics <5 km away), 157 (32%) moved out of the district but stayed in the Cape Town Metropole, 12 (2%) moved out of the region but stayed in the Western Cape and 46 (9%) accessed care in other provinces (Table S1). Figure 2 shows the geographic spread of clinics accessed (A) within the Cape Town Metropole, and (B) across South Africa.

Of 485 women who successfully linked to care after the integrated clinic, 101 women (21%) moved to ≥2 clinics (maximum 3) during 30 months of follow‐up (Figure 1, Table S1). Younger age and being unemployed were associated with moving to ≥2 clinics (aRR 1.10 95% CI 1.02 to 1.18 and aRR 1.06 95% CI 0.99 to 1.12 respectively) (Table S3). Total follow‐up time from ART initiation to the last available clinic visit did not differ by movement frequency (median 28 months in both groups, *p* = 0.787). However, women found at ≥2 clinics were more likely to ever access care outside of Cape Town (38% *vs*. 5%) and had a greater maximum distance between clinics (median 4 km *vs*. 1 km) compared to women who did not move again after linking.

### 3.3 Mobility, retention and HIV VL

Of the 485 women who did have evidence of successfully linking after the integrated clinic, 438 (90%) and 398 (82%) women had evidence of being retained at 12 (6 to <18) and 24 (18 to <30) months after ART initiation respectively (Table S1). Evidence of retention at both 12 and 24 months was found for 363 women (75% of women who linked) (Table 3). When combining those who did not link to care after transfer (n = 132) and those who linked but were subsequently LTFU (n = 122), 71% (n = 438) and 65% (n = 398) of women were retained at 12 and 24 months after ART initiation respectively; 59% (n = 363) of women were retained in care at both time points.

**Table 3:** Description of 485 HIV‐positive women who linked to care after leaving the integrated clinic, by whether they were retained in HIV care at both 12 and 24 months after ART initiation. Presented as n (%) unless specified

	Retained	Not retained	All women	*p*‐value
Number of women	363 (75)	122 (25)	485 (100)	
Characteristics at enrolment				
Mean age (SD)	29 (5.4)	28 (5.2)	29 (5.4)	0.025
Age ≤25	81 (22)	41 (34)	122 (25)	0.013
Married/cohabiting	173 (48)	43 (35)	216 (45)	0.017
Completed secondary school	93 (26)	31 (25)	124 (26)	0.963
Employed	144 (40)	38 (31)	182 (38)	0.093
First pregnancy	56 (15)	27 (22)	83 (17)	0.089
Intended pregnancy	124 (34)	22 (18)	146 (30)	0.001
Diagnosed with HIV in this pregnancy	184 (51)	71 (58)	255 (53)	0.151
Mean weeks gestation (SD)	20 (7.2)	23 (2.6)	21 (4.4)	0.001
Presented for ANC ≤20 weeks	184 (51)	47 (39)	231 (48)	0.020
Median (IQR) CD4 cell count at presentation for ANC (n = 474)	336 (235 to 499)	346 (242 to 537)	342 (235 to 509)	0.269
Characteristics at delivery				
Place of delivery (n = 467)				0.694
Delivered in primary care	141 (40)	49 (42)	190 (41)	
Delivered at tertiary hospital	210 (60)	67 (58)	277 (59)	
Delivery outcome				0.042
Live birth	349 (96)	115 (94)	464 (96)	
Stillbirth	7 (2)	4 (3)	11 (2)	
Miscarriage	6 (2)	0 (0)	6 (1)	
Unknown	1 (<1)	3 (2)	4 (1)	
Characteristics postpartum				
Median (IQR) months from ART initiation until last evidence of accessing care	29 (27 to 29)	15 (9 to 22)	28 (21 to 29)	<0.001
Number of clinics after the integrated clinic				0.015
Attended 1 clinic	278 (77)	106 (87)	384 (79)	
Attended ≥ 2 clinics	85 (23)	16 (13)	101 (21)	
Median furthest distance (km) moved between clinics	1.07 (0.69 to 3.23)	1.07 (0.01 to 7.87)	1.07 (0.69 to 3.23)	0.868
Area moved after integrated clinic				0.252
Same health district	207 (57)	63 (52)	270 (56)	
Cape Town Metropole	117 (32)	40 (33)	157 (32)	
Western Cape Province	10 (3)	2 (2)	12 (2)	
Out of the Western Cape Province	29 (8)	17 (14)	46 (9)	

Retention in care at both 12 and 24 months after ART initiation was associated with attending only one clinic, being >25 years old, being married, being employed, being multigravida, having a planned pregnancy and early presentation for ANC. The association with having a planned pregnancy (aRR 1.20 95% CI 1.09 to 1.33), age >25 years (aRR 1.17 95% CI 1.02 to 1.33) and presenting for ANC ≤20 weeks (aRR 1.10 95% CI 0.99 to 1.21) persisted in multivariable models (Table S3). In sensitivity analyses, having a planned pregnancy was similarly predictive of retention at 24 months after ART initiation and at 18 months postpartum, and the association between being multigravida and being retained maintained a similar effect size but reached statistical significance (Table S3). Distance moved was not associated with being retained in care.

VL measures at least 12 months after ART initiation were available for 338 of 363 women (93%) who were retained in HIV care at both 12 and 24 months after ART initiation (Table S4). There were 273 (81%) and 294 (87%) women who were virally suppressed ≤50 and ≤1000 copies/mL respectively (Tables S4 and S5). Attending ≥2 clinics reduced the likelihood of having a VL ≤50 copies/mL in multivariable models (aRR 0.81 95% CI 0.69 to 0.95); this association was not statistically significant for VL ≤1000 copies/mL (Table 4). Being diagnosed with HIV in the current pregnancy was associated with having a VL ≤50 and ≤1000 copies/mL at least 12 months after ART initiation. Age >25 years predicted VL ≤50 copies/mL, and being married/cohabiting or employed were predictive of having a VL ≤1000 copies/mL in multivariable models (Table 4).

**Table 4:** Poisson regression model among 338 women who were retained in care and had a VL available at least 12 months after ART initiation, predicting (A) VL ≤50 copies/mL at least 12 months after ART initiation, and (B) VL ≤1000 copies/mL at least 12 months after ART initiation (n = 325 with data complete). Presented as unadjusted (RR) and adjusted (aRR) risk ratios with 95% confidence intervals (CI)

	Crude		Adjusted	
	RR	95% CI	aRR	95% CI
(A) VL ≤50 copies/mL at least 12 months after ART initiation				
Attended ≥2 clinics after the integrated clinic	0.80	0.68 to 0.94	0.81	0.69 to 0.95
Age >25	1.18	1.01 to 1.38	1.17	1.01 to 1.36
Completed secondary school	1.11	1.00 to 1.23	–	
Married/cohabiting	1.10	0.99 to 1.21	–	
Planned pregnancy	1.09	0.99 to 1.21	1.10	0.99 to 1.21
Presented for ANC <20 weeks gestation	1.06	0.96 to 1.18	–	
Diagnosed with HIV in this pregnancy	1.15	1.03 to 1.27	1.15	1.04 to 1.28
Employed	1.13	1.02 to 1.25	1.08	0.98 to 1.19
(B) VL ≤1000 copies/mL at least 12 months after ART initiation				
Attended ≥2 clinics after the integrated clinic	0.91	0.81 to 1.02	0.92	0.82 to 1.03
Age >25	1.14	1.00 to 1.29	1.10	0.97 to 1.24
Completed secondary school	1.08	0.99 to 1.17	–	
Married/cohabiting	1.14	1.05 to 1.23	1.14	1.06 to 1.24
Planned pregnancy	1.10	1.02 to 1.19	–	
Presented for ANC <20 weeks gestation	1.08	0.99 to 1.17	–	
Diagnosed with HIV in this pregnancy	1.08	0.99 to 1.17	1.10	1.01 to 1.19
Employed	1.13	1.04 to 1.22	1.12	1.04 to 1.21

## 4 Discussion

This unique study describes outcomes over 30 months of follow‐up for a cohort of women who initiated ART in an integrated ANC and ART clinic and who were required to transfer ART clinics after delivery. Overall, 20% of women did not link to a new clinic within 30 months of ART initiation and an additional 21% were subsequently LTFU after linking. Cumulatively, 41% of women were not retained in care at both 12 and 24 months after ART initiation. Younger women emerged as consistently at risk for not linking to care, non‐retention, non‐suppression and attending ≥2 different clinics.

Our findings add to the limited literature on retention of postpartum women in Africa beyond 12 months on ART 15. Overall retention at 12 and 24 months after ART initiation (71% and 65% respectively) were broadly comparable to reports from other parts of sub‐Saharan Africa. A recent study from Malawi, using a more stringent definition of retention, found that 77% and 71% of women were retained at 12 and 24 months on ART respectively 11. In data from Zimbabwe and Mozambique, only 68% and 42% of women were still receiving ART 12 months after ART initiation 24, 25. A recent systematic review found a pooled estimate of 76% retained at 12 months on ART in African cohorts 15. Reported retention in HIV care is often facility specific. Individuals who are transferred to new clinics are often considered retained in care, censored at the time of transfer or excluded from analyses 26, 27. In contrast, our results are from a cohort where all women were required to transfer care and access to HIV care was traced to any routine primary healthcare clinic in South Africa. It is of concern that, even after tracing women's movement to different clinics, 41% of women were not retained through 12 and 24 months after ART initiation. Importantly, women who never linked to care after the integrated clinic accounted for half the LTFU seen in our cohort. This highlights the need to incorporate support for linkage to care where movement between ART clinics after delivery is required.

Current infant feeding guidelines recommend that HIV‐positive women breastfeed their children for up to 24 months postpartum, making continued postpartum retention critical not only for maternal health and sexual transmission, but also to prevent MTCT in the breastfeeding period 21, 28. Breastfeeding status and access to routine child health services, although not indicators readily available in routine data systems, may impact maternal mobility and engagement in HIV care. In addition, routine child health services are very well attended in many settings and could provide opportunities to re‐engage mothers who fail to link or are LTFU from HIV care.

Despite concerning retention levels, these results showed reassuring levels of viral suppression among women who were retained in care in this cohort. Our results suggest that increased clinic movement could be associated with increased risk of viraemia. However, we were unable to ascertain viral load outcomes for women who were not retained in care and therefore cannot conclusively determine the impact of mobility on HIV viral load. Younger age was a shared risk factor for not linking to care, more frequent mobility, non‐retention and raised VL. This adds to the substantial evidence indicating that younger HIV‐positive women are often at increased risk for poor treatment outcomes 15, 29, 30, 31, 32, 33. Although associations were small, younger age, timing of presentation for ANC and relationship status could flag women requiring additional support to link to care postpartum. Primigravity and unplanned pregnancy, previously linked to adverse maternal and child outcomes 34, 35, may also flag women requiring targeted retention interventions. Although the impact of pregnancy intention on long‐term ART outcomes is not clear, optimizing family planning services for both HIV‐positive and negative women remains a vital component of strategies to prevent MTCT and improve maternal and child health. Importantly, interventions are needed not only at ART initiation facilities but also beyond the facility to promote continued retention in care when mobility is necessary.

Women accessed care at a variety of different clinics. Although some clinics provide combined appointments for HIV‐positive mothers and their children, many require different appointments for maternal ART and routine child health and quite often these services are offered at different clinics. We were unable to systematically assess each model of care but this should be a consideration in future work. The clinic movement seen in this analysis has further implications for linking mother‐child pairs and monitoring long‐term child and maternal outcomes. In both routine programmes and research cohorts, retention in ART care is frequently based on whether an individual is still receiving care from the clinic at which they started treatment 36, 37, 38. A review of studies in low‐ and middle‐income countries that actively traced patients to ascertain their status, showed a pooled estimate of 19% of adults considered LTFU were continuing care at other clinics 39. Although the most recent World Health Organization recommendations for monitoring include using unique patient identifiers to allow linkage across health services, this is not a reality in many settings 40. Availability of facility‐linked data and the choice of data sources used will impact the ability to monitor long‐term outcomes of women on lifelong ART and their children 41.

The results of this analysis should be interpreted with the following additional caveats in mind. Although a strength of this study is the availability of diverse data sources throughout the Western Cape Province and nationally for evaluating evidence of engagement in care, not all contacts with the health system are captured into routine electronic databases which could lead to underestimation of retention. Attempts were made to ascertain vital status using clinic records but unknown deaths may contribute to non‐linkage and non‐retention. Both a strength and limitation is that these results are applicable to a cohort of women required to transfer their ART care after delivery. We were unable to classify additional mobility after linkage and outcomes may vary between formal clinic transfers and patient‐initiated mobility.

Another important limitation of this analysis is that the same data sources were used to define mobility and retention in care and the mobility patterns among women who were not observed to be in care cannot be known. Surprisingly, employment was not strongly associated with mobility or any of the outcomes in this study 2, 42. This is likely a limitation of the measure which only assessed employment status at entry into ANC. Women may have returned to or started work after delivery with possible impacts on both mobility and HIV care access. Studies which can assess mobility independently from access to routine health services and which can assess changing risk factors such as employment and relationship status over time, will be required to further understand postpartum mobility and the impact on ART outcomes.

These data add important insights regarding mobility and retention in care in postpartum women up to 30 months after ART initiation in pregnancy. The step of moving care between clinics is a vulnerable step in the HIV care continuum and even women who manage to link successfully, particularly younger, unmarried women and those who present late for ANC, remain vulnerable to subsequent LTFU and viraemia. Models of care to provide ART to pregnant and postpartum women need to accommodate women's mobility and where they choose to access care. Facility‐based interventions may not be sufficient to support postpartum retention. For example, South African differentiated models of care and mHealth interventions are starting to provide non‐facility based support for mothers 43, 44. Further consideration is needed on how continuous support for engagement in care can be provided as mothers living with HIV continue with their daily lives after delivery.

## 5 Conclusions

We found that less than two‐thirds of women who started ART in an integrated ANC‐ART clinic were retained in care at both 12 and 24 months after ART initiation. Losses occurred at the initial mandatory postpartum transfer and after successful linkage to care. Women who linked to care attended a wide range of facilities creating challenges for monitoring postpartum outcomes. Based on these data there is a clear and urgent need for interventions that extend outside of health facilities to help support postpartum women, and young mothers in particular, to remain engaged in lifelong ART care.

## Competing interests

The authors declare no competing interests.

## Authors’ contributions

TP conceived the design, conducted the analysis and drafted the manuscript. KC contributed to the study design and analysis. AZ, CO, EJA and LM contributed to the study design and manuscript writing. All authors read and approved the final manuscript.

## Funding

This research was supported by the President's Emergency Plan for AIDS Relief (PEPFAR) through the National Institute of Child Health and Human Development (NICHD), Grant Number R01HD074558. Dr Clouse was supported by the National Institute of Mental Health (K01MHH107256). We thank all participating mothers and the research team in Gugulethu.

## Supporting information


**Table S1.** Description of 485 HIV‐positive women, who had evidence of linking to care after leaving the integrated clinic, by the number of different clinics attended up to 30 months after ART initiation
**Table S2.** Poisson regression model among 485 women who linked to care after the integrated clinic, predicting whether women moved to more than one additional clinic
**Table S3.** Poisson regression model among 485 women who linked to care after the integrated clinic, predicting (A) retention in care at 12 and 24 months after ART initiation, (B) retention in care at 24 months after ART initiation, and (C) retention in care at 18 months postpartum
**Table S4.** Description of 338 HIV‐positive women with viral load (VL) ≤50 and >50 copies/mL who were retained at both 12 and 24 months after ART initiation and had a VL available at least 12 months after ART initiation
**Table S5.** Description of 338 HIV‐positive women with viral load (VL) ≤1000 and >1000 copies/mL who were retained at both 12 and 24 months after ART initiation and had a VL available at least 12 months after ART initiation
**Table S6.** Description of study outcomes by the design of follow up received in the parent MCH‐ART study
