# Supplementary material for: Linkage to care, mobility and retention of HIV‐positive postpartum women in antiretroviral therapy services in South Africa
Source: J Int AIDS Soc. 2018 Jul 19;21(Suppl Suppl 4):e25114. doi: 10.1002/jia2.25114 (PMC6053482; doi:10.1002/jia2.25114)
Supplement: Supplementary file 1 — Table S1. Description of 485 HIV‐positive women, who had evidence of linking to care after leaving the integrated clinic, by the number of different clinics attended up to 30 months after ART initiation Table S2. Poisson regression model among 485 women who linked to care after the integrated clinic, predicting whether women moved to more than one additional clinic Table S3. Poisson regression model among 485 women who linked to care after the integrated clinic, predicting (A) retention in care at 12 and 24 months after ART initiation, (B) retention in care at 24 months after ART initiation, and (C) retention in care at 18 months postpartum Table S4. Description of 338 HIV‐positive women with viral load (VL) ≤50 and >50 copies/mL who were retained at both 12 and 24 months after ART initiation and had a VL available at least 12 months after ART initiation Table S5. Description of 338 HIV‐positive women with viral load (VL) ≤1000 and >1000 copies/mL who were retained at both 12 and 24 months after ART initiation and had a VL available at least 12 months after ART initiation Table S6. Description of study outcomes by the design of follow up received in the parent MCH‐ART study [file JIA2-21-e25114-s001.docx]

Appendix: Supplementary (S) tables:

Table S1. Description of 485 HIV-positive women, who had evidence of linking to care after leaving the integrated clinic, by the number of different clinics attended up to 30 months after ART initiation. Presented as n (%) unless specified.

|  | **Attended 1 clinic** | **Attended ≥2 clinics** | **All women** | **p-value** |
| --- | --- | --- | --- | --- |
| Number of women | 384 (79) | 101 (21) | 485 (100) |  |
| **Characteristics at enrolment** |  |  |  |  |
| Mean age (SD) | 29 (5.3) | 28 (5.3) | 28 (25-32) | 0.034 |
| Age ≤25 | 86 (22) | 36 (36) | 122 (25) | 0.006 |
| Married/cohabiting | 169 (44) | 47 (47) | 216 (45) | 0.650 |
| Completed secondary school | 104 (27) | 20 (20) | 124 (26) | 0.136 |
| Employed | 152 (40) | 30 (30) | 182 (38) | 0.068 |
| First pregnancy | 61 (16) | 22 (22) | 83 (17) | 0.161 |
| Intended pregnancy | 114 (30) | 32 (32) | 146 (30) | 0.697 |
| Diagnosed with HIV in this pregnancy | 199 (52) | 56 (55) | 255 (53) | 0.516 |
| Mean weeks gestation (SD) | 21 (7.4) | 21 (7.4) | 21 (7.4) | 0.373 |
| Presented for ANC ≤20 weeks | 183 (48) | 48 (48) | 231 (48) | 0.981 |
| Median (IQR) CD4 cell count at presentation for ANC (n=481) | 341 (236-522) | 343 (211-496) | 341 (235-509) | 0.344 |
| **Characteristics at delivery** |  |  |  |  |
| Place of delivery (n=467) |  |  |  |  |
| Delivered in primary care | 150 (40) | 40 (42) | 190 (41) | 0.826 |
| Delivered at tertiary hospital | 221 (60) | 56 (58) | 277 (59) |  |
| Delivery outcome |  |  |  |  |
| Live birth | 369 (96) | 95 (94) | 464 (96) | 0.486 |
| Stillbirth | 8 (2) | 3 (3) | 11 (2) |  |
| Miscarriage | 5 (1) | 1 (1) | 6 (1) |  |
| Unknown | 2 (1) | 2 (2) | 4 (1) |  |
| **Characteristics postpartum** |  |  |  |  |
| Median (IQR) months from ART initiation until last evidence of accessing care | 28 (21-29) | 28 (22-29) | 28 (21-29) | 0.787 |
| Median furthest distance (km) moved between clinics (SD) | 1.06 (0.01-2.55) | 4.14 (1.73-863.78) | 1.07 (0.69-3.23) | <0.001 |
| Area moved after integrated clinic |  |  |  |  |
| Same health district | 231 (60) | 39 (39) | 270 (56) | <0.001 |
| Cape Town Metropole | 133 (35) | 24 (24) | 157 (32) |  |
| Western Cape Province | 5 (1) | 7 (7) | 12 (2) |  |
| Out of the Western Cape Province | 15 (4) | 31 (31) | 46 (9) |  |
| Retention in HIV care after ART initiation |  |  |  |  |
| Retained at 12 months | 340 (89) | 98 (97) | 438 (90) | 0.010 |
| Retained at 24 months | 310 (81) | 88 (87) | 398 (82) | 0.136 |
| Retained at 12 and 24 months | 278 (72) | 85 (84) | 363 (75) | 0.015 |
| Retained at 18 months after delivery | 315 (82) | 90 (89) | 405 (84) | 0.088 |

Table S2. Poisson regression model among 485 women who linked to care after the integrated clinic, predicting whether women moved to more than one additional clinic. Presented as unadjusted (RR) and adjusted (aRR) risk ratios with 95% confidence intervals (CI).

|  | **Crude** | | | **Adjusted** | | |
| --- | --- | --- | --- | --- | --- | --- |
|  | **RR** | **95% CI** |  | **aRR** | **95% CI** |  |
| Age ≤25 | 1.09 | 1.02-1.18 |  | 1.10 | 1.02-1.18 |  |
| Not employed | 1.06 | 1.00-1.13 |  | 1.06 | 0.99-1.12 |  |

Table S3. Poisson regression model among 485 women who linked to care after the integrated clinic, predicting A) retention in care at 12 and 24 months after ART initiation, B) retention in care at 24 months after ART initiation, and C) retention in care at 18 months postpartum. Presented as unadjusted (RR) and adjusted (aRR) risk ratios with 95% confidence intervals (CI).

|  | **Crude** | | | **Adjusted** | | |  |
| --- | --- | --- | --- | --- | --- | --- | --- |
| **A: retention in care at 12 and 24 months after ART initiation** | | | | | | | |
|  | **RR** | **95% CI** |  | **aRR** | **95% CI** |  |  |
| Age >25 | 1.17 | 1.02-1.34 |  | 1.17 | 1.02-1.33 |  |  |
| Married/cohabiting | 1.13 | 1.02-1.26 |  | - |  |  |  |
| Primigravida | 0.88 | 0.75-1.04 |  | - |  |  |  |
| Planned pregnancy | 1.20 | 1.09-1.33 |  | 1.20 | 1.09-1.33 |  |  |
| Presented for ANC <20 weeks gestation | 1.13 | 1.02-1.25 |  | 1.10 | 0.99-1.21 |  |  |
| Employed | 1.09 | 0.98-1.21 |  | - |  |  |  |
| **B: retention in care at 24 months after ART initiation** | | | | | | | |
|  | **RR** | **95% CI** |  | **aRR** | **95% CI** |  |  |
| Age >25 | 1.06 | 0.95-1.17 |  | - |  |  |  |
| Married/cohabiting | 1.07 | 0.99-1.16 |  | - |  |  |  |
| Primigravida | 0.89 | 0.78-1.02 |  | 0.87 | 0.76-0.99 |  |  |
| Planned pregnancy | 1.15 | 1.07-1.24 |  | 1.17 | 1.08-1.27 |  |  |
| Presented for ANC <20 weeks gestation | 1.08 | 0.99-1.17 |  | - |  |  |  |
| Employed | 0.92 | 0.85-1.00 |  | 1.07 | 0.99-1.16 |  |  |
| **C: retention in care at 18 months postpartum** | | | | | | | |
|  | **RR** | **95% CI** |  | **aRR** | **95% CI** |  |  |
| Age >25 | 1.07 | 0.97-1.18 |  | - |  |  |  |
| Married/cohabiting | 1.07 | 0.99-1.16 |  | - |  |  |  |
| Primigravid | 0.89 | 0.78-1.01 |  | 0.87 | 0.77-0.99 |  |  |
| Planned pregnancy | 1.14 | 1.06-1.22 |  | 1.16 | 1.07-1.25 |  |  |
| Presented for ANC <20 weeks gestation | 1.06 | 0.98-1.15 |  | - |  |  |  |
| Employed | 1.04 | 0.93-1.13 |  | - |  |  |  |

Table S4. Description of 338 HIV-positive women with viral load (VL) ≤50 and >50 copies/ml who were retained at both 12 and 24 months after ART initiation and had a VL available at least 12 months after ART initiation. Presented as n (%) unless specified.

|  | **VL ≤50 copies/ml** | **VL >50 copies/ml** | **All women** | **p-value** |
| --- | --- | --- | --- | --- |
| Number of women | 273 (81) | 65 (19) | 338 (100) |  |
| VL source |  |  |  |  |
| NHLS | 174 (64) | 32 (49) | 206 (61) | 0.031 |
| MCH-ART study | 99 (36) | 33 (51) | 132 (39) |  |
| Median (IQR) months postpartum at time of VL test | 18 (18-20) | 18 (17-21) | 18 (18-20) | 0.933 |
| Median months since ART start at time of VL test | 23 (22-24) | 23 (21-24) | 23 (21-24) | 0.449 |
| **Characteristics at enrolment** |  |  |  |  |
| Mean age (SD) | 30 (5.4) | 27 (4.8) | 29 (5.4) | 0.003 |
| Age ≤25 | 53 (19) | 22 (34) | 75 (22) | 0.012 |
| Married/cohabiting | 133 (49) | 24 (37) | 157 (46) | 0.087 |
| Completed secondary school | 74 (27) | 11 (17) | 85 (25) | 0.089 |
| Employed | 117 (43) | 18 (28) | 135 (40) | 0.025 |
| First pregnancy | 39 (14) | 13 (20) | 52 (15) | 0.251 |
| Intended pregnancy | 101 (37) | 17 (26) | 118 (35) | 0.099 |
| Diagnosed with HIV in this pregnancy | 149 (55) | 24 (37) | 173 (51) | 0.010 |
| Mean weeks gestation (SD) | 21 (8.9) | 20 (7.1) | 20 (7.2) | 0.118 |
| Presented for ANC ≤20 weeks gestation | 143 (52) | 29 (45) | 172 (51) | 0.260 |
| Median (IQR) CD4 cell count at presentation for ANC (n=330) | 341 (235-520) | 350 (232-440) | 337 (235-504) | 0.525 |
| **Characteristics at delivery** |  |  |  |  |
| Place of delivery (n=327) |  |  |  |  |
| Delivered in primary care | 109 (41) | 25 (41) | 134 (41) | 0.999 |
| Delivered at tertiary hospital | 157 (59) | 36 (59) | 193 (59) |  |
| Delivery outcome |  |  |  |  |
| Live birth | 263 (96) | 63 (97) | 326 (96) | 0.131 |
| Stillbirth | 4 (1) | 1 (2) | 5 (1) |  |
| Miscarriage | 6 (2) | 0(0) | 6 (2) |  |
| Unknown | 0 (0) | 1 (2) | 1 (<1) |  |
| **Characteristics postpartum** |  |  |  |  |
| Number of clinics after the integrated clinic |  |  |  |  |
| Attended 1 clinic | 221 (81) | 40 (62) | 261 (77) | 0.001 |
| Attended ≥ 2 clinics | 52 (19) | 25 (38) | 77 (23) |  |
| Median furthest distance (km) moved between clinics | 1.06 (0.69-3.04) | 1.99 (0.71-3.23) | 1.06 (0.69-3.23) | 0.110 |
| Area moved after integrated clinic |  |  |  |  |
| Same health district | 160 (58) | 35 (54) | 195 (57) | 0.508 |
| Cape Town Metropole | 89 (33) | 21 (32) | 110 (33) |  |
| Western Cape Province | 6 (2) | 3 (5) | 9 (3) |  |
| Out of the Western Cape Province | 118 (7) | 6 (9) | 24 (7) |  |

Table S5. Description of 338 HIV-positive women with viral load (VL) ≤1000 and >1000 copies/ml who were retained at both 12 and 24 months after ART initiation and had a VL available at least 12 months after ART initiation. Presented as n (%) unless specified.

|  | **VL ≤1000 copies/ml** | **VL >1000 copies/ml** | **All women** | **p-value** |
| --- | --- | --- | --- | --- |
| Number of women | 294 (87) | 44 (13) | 338 (100) |  |
| **Characteristics at enrolment** |  |  |  |  |
| Mean age (SD) | 29 (5.4) | 27 (5.0) | 29 (5.4) | 0.010 |
| Age ≤25 | 59 (20) | 16 (36) | 75 (22) | 0.015 |
| Married/cohabiting | 146 (50) | 11 (25) | 157 (46) | 0.002 |
| Completed secondary school | 78 (27) | 7 (16) | 85 (25) | 0.130 |
| Employed | 126 (43) | 9 (20) | 135 (40) | 0.005 |
| First pregnancy | 42 (14) | 10 (23) | 52 (15) | 0.148 |
| Intended pregnancy | 109 (37) | 9 (20) | 118 (35) | 0.031 |
| Diagnosed with HIV in this pregnancy | 156 (53) | 17 (39) | 173 (51) | 0.074 |
| Mean weeks gestation (SD) | 21 (8.9) | 23 (7.0) | 20 (7.2) | 0.012 |
| Presented for ANC <20 weeks gestation | 155 (53) | 17 (39) | 172 (51) | 0.081 |
| Median (IQR) CD4 cell count at presentation for ANC (n=328) | 345 (237-512) | 320 (200-436) | 337 (235-504) | 0.253 |
| **Characteristics at delivery** |  |  |  |  |
| Place of delivery (n=327) |  |  |  |  |
| Delivered in primary care | 118 (42) | 16 (37) | 134 (41) | 0.590 |
| Delivered at tertiary hospital | 166 (58) | 27 (63) | 193 (59) |  |
| Delivery outcome |  |  |  |  |
| Live birth | 283 (96) | 43 (98) | 326 (96) | 0.736 |
| Stillbirth | 4 (1) | 1 (1) | 5 (1) |  |
| Miscarriage | 6 (2) | 0 (0) | 6 (2) |  |
| Unknown | 1 (<1) | 0 (0) | 1 (<1) |  |
| **Characteristics postpartum** |  |  |  |  |
| Number of clinics after the integrated clinic |  |  |  |  |
| Attended 1 clinic | 232 (79) | 29 (66) | 261 (77) | 0.055 |
| Attended ≥ 2 clinics | 62 (21) | 15 (34) | 77 (23) |  |
| Area moved after integrated clinic |  |  |  |  |
| Same health district | 170 (58) | 25 (57) | 195 (57) | 0.398 |
| Cape Town Metropole | 94 (32) | 16 (36) | 110 (33) |  |
| Western Cape Province | 7 (3) | 2 (5) | 19 (3) |  |
| Out of the Western Cape Province | 23 (8) | 1 (2) | 24 (7) |  |

Table S6. Description of study outcomes by the design of follow up received in the parent MCH-ART study.

|  | **Not enrolled in the MCH-ART trial**  **(no prospective follow-up after delivery)** | **Enrolled in the MCH-ART trial and received standard transfer out of the integrated clinic** | **Enrolled in the MCH-ART trial and received delayed transfer out of the integrated clinic** |
| --- | --- | --- | --- |
| Number of women | 152 | 236 | 229 |
| Median months of follow-up after leaving the integrated clinic | 26 (24-27) | 25 (24-26) | 18 (14-23) |
| Linked to care | 122 (80) | 193 (82) | 170 (74) |
| If linked (n=485), attended ≥2 clinics | 28 (23) | 32 (17) | 41 (24) |
| Retained in care at 12 months on ART | 106 (70) | 176 (75) | 193 (84) |
| Retained in care at 24 months on ART | 95 (63) | 149 (63) | 158 (69) |
| Retained at both 12 and 24 months on ART | 80 (53) | 136 (58) | 150 (66) |
| Number of women retained in care at both 12 and 24 months on ART and with viral load after 12 months on ART available (n=341) | 62 (41) | 132 (56) | 147 (64) |
| Viral load ≤50 copies/ml | 52 (84) | 104 (79) | 119 (81) |
| Viral load ≤1000 copies/ml | 57 (92) | 113 (86) | 126 (86) |
